# Analysis of the Neuroproteome Associated With Cell Therapy After Intranigral Grafting in a Mouse Model of Parkinson Disease

**DOI:** 10.3389/fnins.2021.621121

**Published:** 2021-03-11

**Authors:** Hassan Dakik, Sarah Mantash, Ali Nehme, Firas Kobeissy, Masoud Zabet-Moghaddam, Parvin Mirzaei, Yehia Mechref, Afsaneh Gaillard, Laetitia Prestoz, Kazem Zibara

**Affiliations:** ^1^ER045, PRASE, Lebanese University, Beirut, Lebanon; ^2^Université de Tours, Tours, France; ^3^INSERM, Laboratoire de Neurosciences Expérimentales et Cliniques, Université de Poitiers, Poitiers, France; ^4^McGill University and Génome Québec Innovation Centre, McGill University, Montreal, QC, Canada; ^5^Department of Biochemistry and Molecular Genetics, Faculty of Medicine, American University of Beirut, Beirut, Lebanon; ^6^Center for Biotechnology and Genomics, Texas Tech University, Lubbock, TX, United States; ^7^Department of Biology, Faculty of Sciences-I, Lebanese University, Beirut, Lebanon

**Keywords:** proteome, cell therapy, nigrostriatal pathway, transplantation, Parkinson disease, systems biology, translational animal models, neurodegenerative disorders (ND)

## Abstract

Advances in large-scale proteomics analysis have been very useful in understanding pathogenesis of diseases and elaborating therapeutic strategies. Proteomics has been employed to study Parkinson disease (PD); however, sparse studies reported proteome investigation after cell therapy approaches. In this study, we used liquid chromatography–tandem mass spectrometry and systems biology to identify differentially expressed proteins in a translational mouse model of PD after cell therapy. Proteins were extracted from five nigrostriatal-related brain regions of mice previously lesioned with 6-hydroxydopamine in the substantia nigra. Protein expression was compared in non-grafted brain to 1 and 7 days after intranigral grafting of E12.5 embryonic ventral mesencephalon (VM). We found a total of 277 deregulated proteins after transplantation, which are enriched for lipid metabolism, oxidative phosphorylation and PD, thus confirming that our animal model is similar to human PD and that the presence of grafted cells modulates the expression of these proteins. Notably, seven proteins (Acta1, Atp6v1e1, Eci3, Lypla2, Pip4k2a, Sccpdh, and Sh3gl2) were commonly down-regulated after engraftment in all studied brain regions. These proteins are known to be involved in the formation of lipids and recycling of dopamine (DA) vesicle at the synapse. Moreover, intranigral transplantation of VM cells decreased the expression of proteins related to oxidative stress, especially in the nigrostriatal pathway containing the DA grafted neurons. In the same regions, an up-regulation of several proteins including α-synuclein and tyrosine hydroxylase was observed, whereas expression of tetraspanin 7 was shut down. Overall, these results suggest that intranigral transplantation of VM tissue in an animal model of PD may induce a decrease of oxidative stress in the nigrostriatal pathway and a restoration of the machinery of neurotransmitters, particularly DA release to promote DA transmission through a decrease of D2 DA receptors endocytosis. Identification of new mechanistic elements involved in the nigrostriatal reconstruction process, using translational animal models and systems biology, is a promising approach to enhance the repair of this pathway in PD patients undergoing cell therapy.

## Introduction

Parkinson disease (PD) is a progressive neurodegenerative disorder characterized by the degeneration of the dopamine (DA) nigrostriatal system, affecting 10 million people worldwide (Delenclos et al., 2016). The major neuropathological features of PD are the loss of dopaminergic neurons within the substantia nigra (SN) pars compacta (SNpc) and deposition of α-synuclein aggregates, the Lewy bodies, within the cytoplasm of DA neurons (Kalia and Lang, 2015). Several studies have elucidated some underlying mechanisms associated with the onset and progression of PD, which include four major events: α-synuclein protein accumulation, mitochondrial deficits, oxidative stress, and impairments in intracellular trafficking (Poewe et al., 2017). Indeed, toxic forms of α-synuclein lead to their aggregation into insoluble fibrils constituting the Lewy pathology (Kim and Lee, 2008). This would lead to the interruption of several proteasomal and lysosomal degradation pathways including macroautophagy, chaperon-mediated autophagy, and ubiquitin–proteasome systems (Xilouri et al., 2009; Emmanouilidou et al., 2010; Tanik et al., 2013). In addition, Lewy bodies increase oxidative stress and inflammatory pathways leading to neuronal cell death (Poewe et al., 2017). Moreover, mitochondrial defects are associated with the pathogenesis of PD (Schapira, 2007; Bose and Beal, 2016; Franco-Iborra et al., 2016). For instance, the mitochondrial serine–threonine protein kinase PINK-1, parkin (E3 ubiquitin ligase), and the deglycase and chaperone protein DJ-1 are implicated in the degradation of impaired mitochondria to maintain cell survival through mitophagy. Mutations in these genes are highly associated with mitochondrial dysfunction in PD (Pickrell and Youle, 2015). Consequently, oxidative damage occurs in the nigral DA neurons of PD patients (Jenner, 2003), resulting into prominent deficits in the autophagy lysosomal pathways (Dias et al., 2013).

Several transcriptional studies were performed to identify the molecular pathways implicated in PD initiation and development. However, differential expression at the RNA level may not reflect the functional aspect defined by the proteins within a tissue. Thus, the development of proteomics tools, such as high throughput mass-spectrometry, has improved our understanding of diseases such as PD, by accelerating the discovery of new candidate targets and revealing functional pathways involved in the pathogenesis of this disease. Basso et al. (2003) were the first to publish a proteome analysis of mesencephalic tissues of patients with idiopathic PD. Thereafter, a number of proteomic studies have been conducted on brain tissue, blood, and cerebrospinal fluid of PD patients and have identified a wide array of protein alterations underlying disease pathogenesis (Dixit et al., 2019, for review). Proteomics was also undertaken in cellular and animal models of PD (Kasap et al., 2017). For instance, proteomic reports identified modulation in the expression of several proteins including calmodulin, cytochrome C, and cytochrome C oxidase in rat brain samples unilaterally treated with 6-hydroxydopamine (6-OHDA) into the medial forebrain bundle (MFB) (Pierson et al., 2004). Other proteomic reports revealed differential expression of β-actin and α-enolase proteins in the SN and the striatum, respectively (De Iuliis et al., 2005). In addition, an increase in the expression of proteins involved in mitochondrial metabolism, antioxidative reaction, and cytoskeleton rearrangements were also demonstrated (Lessner et al., 2010). More recently, mitochondrial proteomes analyzed at 4 days and 4 weeks after 6-OHDA injection in the MFB displayed changes in the expression of proteins involved in oxidative phosphorylation, structural remodeling, cytoskeleton rearrangement, organelle trafficking, axon outgrowth, and regeneration (Kuter et al., 2016).

Regardless of advances in the understanding of PD pathology, it remains challenging to extrapolate this progress into therapeutic strategies. Innovative approaches are still needed to establish effective treatments for PD patients. Proteomics studies conducted in PD may offer a new strategy to better identify novel diagnostic biomarkers, as well as therapeutic approaches related to the development and progression of the disease. Previous studies, including ours (Gaillard et al., 2009; Thompson et al., 2009; Gaillard and Jaber, 2011), showed that grafting of fetal DA cells from ventral mesencephalon (VM) in the lesioned adult mouse SN leads to anatomical and functional recovery of the nigrostriatal circuitry. Indeed, axonal projections of the grafted DA neurons navigate through intermediate targets in the diencephalon to innervate the striatum in the telencephalon, therefore regenerating the damaged DA network. This supports the concept that the host brain tissue is permissive to long axonal navigation and implies that certain molecules, residing in regions surrounding the nigrostriatal pathway, orchestrate the axonal steering of the grafted DA fetal cells to ensure proper DA connectivity, and restore motor performance. Indeed, we have previously shown a modification in the transcriptional expression of axon guidance molecules in the graft and along the nigrostriatal pathway during its reconstruction after transplantation (Kalaani et al., 2016). Therefore, we hypothesize that the expression of specific category of proteins could be modified after transplantation during the nigrostriatal reconstruction between days 1 and 7 after grafting. Moreover, we expect modifications of expression of proteins involved in the dopaminergic machinery as we previously showed that transplantation of dopaminergic cells leads to a functional restoration of this pathway.

In this study, we aimed to clarify the molecular events underlying this anatomical and functional reconstruction and particularly to identify the proteins involved in this model of cell therapy. To this end, we used liquid chromatography–tandem mass spectrometry (LC-MS/MS) to reveal the brain proteome after intranigral grafting of fetal DA neurons in the previously described 6-OHDA–lesioned mouse model of PD (Gaillard et al., 2009). C57BL/6 mice were first lesioned unilaterally with an injection of 6-OHDA into the SN. Mice were then transplanted with VM cell suspension of E12.5 embryos, and five nigrostriatal-related brain regions were collected 1 or 7 days posttransplantation. We performed differential-expression analysis to identify top deregulated proteins and implemented weighted gene coexpression network analysis (WGCNA) to find networks of intercorrelated proteins that are associated with tissue regeneration. Our results showed that differentially expressed proteins were involved in pathways implicated in local inflammatory reactions, oxidative stress, translation, and mitochondrial metabolic pathways. In addition, we observed deregulation of proteins involved in the neurotransmission of DA synapses.

## Materials and Methods

### Animals

All animal experimental procedures were performed in agreement with the guidelines of the French Agriculture and Forestry Ministry (Decree 87849) and approved by the European Communities Council Directive (86/609/EEC). All procedures were approved by the local ethical committee, and efforts were made to reduce the number of animals used and their suffering.

Adults (4–6 months old) C57BL/6 mice were used as well as VM of E12.5 embryos from C57/Bl6 transgenic mice overexpressing enhanced green fluorescent protein (EGFP) under the control of the tyrosine-hydroxylase promoter (C57BL/6JRj TH-EGFP). VMs of embryos were used as a source of cells for transplantation as previously described (Gaillard et al., 2009).

### Lesion With 6-OHDA and Cell Transplantation

**FIGURE 1:**
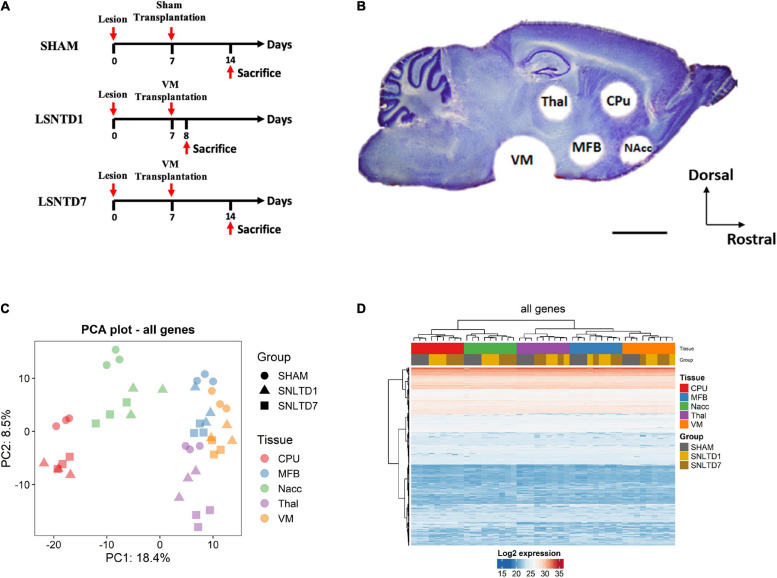
Unsupervised analysis of all proteins in the dataset. **(A)** Schematic representation for the time course of the procedure. The lesion was performed unilaterally with an injection of 6-hydroxydopamine (6-OHDA) in the SNpc to specifically kill dopaminergic neurons. One week following the lesion, VMs from TH-EGFP E12.5 transgenic mice embryos were dissociated, and a cell suspension of 1.5 μL containing 150,000 cells was injected into the lesioned SN. The sham grafted group received only a vehicle solution. Animals were sacrificed at 1 day (LSNTD1, *n* = 3) or 7 days (LSNTD7, *n* = 3) after transplantation. Sham-transplanted mice were sacrificed 7 days after the surgery (SHAM, *n* = 3). **(B)** Punched regions on a sagittal section of adult mouse brain labeled with Nissl stain. CPu, NAcc, MFB, and Thal regions were collected using a 1 mm-diameter punch, whereas VM was collected using a 2 mm-diameter punch (scale bar 2 mm). CPu, caudate putamen; NAcc, nucleus accumbens; Thal, thalamus; MFB, medial forebrain bundle; VM, ventral mesencephalon. Adapted from Kalaani et al. (2016). **(C)** Principal component analysis (PCA) showing the distribution of samples according to PC1 and PC2 that together explain ∼27% of variability between samples. Tissues are presented as different colors, whereas groups are presented as shapes. **(D)** Heatmap showing unsupervised clustering of both genes and samples.

C57BL/6 wild-type mice (*n* = 9) were lesioned unilaterally with an injection of 6-OHDA in the SN to specifically kill dopaminergic cells as previously described (Gaillard et al., 2009), with slight modifications. Briefly, mice were first anesthetized by an intraperitoneal injection of ketamine/xylazine (100 and 10 mg/kg, respectively). One microliter of 6-OHDA (8 μg/μL, Sigma), dissolved in a sterile saline solution containing 0.1% ascorbic acid, was injected into the left SN pars compacta at the following coordinates: AP = −3.2 mm (from bregma), ML = 1.4 mm (from midline), and DV = 3.8 mm (from dura). One week following the lesion, VMs from TH-EGFP E12.5 transgenic embryos were dissociated in 0.6% glucose saline solution, and a cell suspension of 1.5 μL containing 150,000 cells was injected into the lesioned SN mice. The sham grafted group received only a vehicle solution. Time course of the procedure is described in Figure 1A.

### Tissue Processing and Sampling

Animals were sacrificed by cervical dislocation at 1 day [LSNTD1 (lesioned and transplanted SN at day 1), *n* = 3] or 7 days (LSNTD7, *n* = 3) after transplantation. Sham-transplanted mice were sacrificed 7 days after the surgery (sham, *n* = 3). The time points of 1 and 7 days after intranigral transplantation were chosen on the basis of our previously published work. Indeed, 1 day after transplantation, the graft is well-integrated, and fibers begin to grow out. These fibers follow the host nigrostriatal pathway and reinnervate the dorsolateral striatum starting from the seventh day after grafting (Gaillard et al., 2009). Furthermore, variations in the transcript expression of several axon guidance molecules have been demonstrated at 1 and 7 days after intranigral transplantation in five different regions of the nigrostriatal pathway (Kalaani et al., 2016).

The brains were instantly collected and frozen on dry ice and cut into 200 μm sagittal sections using a cryostat. For each animal, five brain regions were collected according to our previous protocol (Kalaani et al., 2016): the caudate putamen (CPu), the nucleus accumbens (NAcc), the MFB, the thalamus (Thal), and the VM. Regions were collected using a 1 mm-diameter puncher for the CPu, NAcc, MFB, and Thal, whereas a 2 mm-diameter puncher was used for the VM region, as illustrated in Figure 1B.

### Protein Extraction and Digestion

Tissue samples were washed with 50 mM ammonium bicarbonate (ABC) buffer. Beads beater (Beadbug microtube homogenizer; Benchmark Scientific, Edison, NJ) was applied to perform cell lysis. Tissues were mixed with 5% sodium deoxycholate (SDC) (Sigma–Aldrich, St. Louis, MO) solution and 3 mm zirconium beads (OPS Diagnostics, Lebanon, NJ) in a 2 mL microtube. The solution of 5% SDC was added for efficient protein extraction. The samples were homogenized at 4°C at 4,000 revolutions/min for 30 s, followed by a 30 s pause. This step was repeated for six times. Next, tissue lysate was sonicated for 30 min in 0°C ice-water bath to enhance the protein dissolving. After centrifuging at 21,100 *g* for 10 min, the supernatant was collected and diluted 10 times by adding 50 mM ABC buffer. Protein concentration was determined by BCA protein assay kit (Thermo Fisher Scientific/Pierce, Rockford, IL) following the manufacturer’s instruction.

A 25 μg aliquot of extracted proteins of each sample was then subjected to reduction, alkylation, and tryptic digestion. ABC (50 mM)–SDC (0.5%) solution was first added to samples to maintain a volume of 50 μL. Proteins were thermally denatured at 80°C for 10 min. The reduction of proteins was performed by adding a 1.25 μL aliquot of 200 mM dithiothreitol (DTT) solution and incubating at 60°C for 45 min. The reduced proteins were then alkylated by adding a 5 μL aliquot of 200 mM iodoacetamide (IAA) solution and incubation at 37°C in the dark for 45 min. To quench the excessive IAA, a 1.25 μL aliquot of DTT solution was added again, and samples were incubated at 37°C for 30 min. Following the reduction and alkylation of proteins, trypsin (Promega, Madison, WI) was added at a ratio of 1:25 (enzyme: proteins, wt/wt) into samples and incubated at 37°C for 18 h. After incubation, formic acid (FA) was added at a final concentration of 0.5% (vol/vol) for the purposes of both quenching the enzymatic reaction and removing the SDC detergent. Samples were then mixed thoroughly and centrifuged at 21,100 *g* for 10 min. The supernatant was collected, SpeedVac dried and resuspended in aqueous solution containing 2% acetonitrile (ACN) and 0.1% FA prior to LC-MS/MS analysis.

### LC-MS/MS Measurement

Aliquots of 1 μg tryptic digests were subjected to the untargeted proteomic analysis. A Dionex 3000 Ultimate nano-LC (Dionex, Sunnyvale, CA) interfaced to an LTQ Orbitrap Velos mass spectrometer (Thermo Fisher Scientific, San Jose, CA) that is equipped with ESI source was used for the analysis. Tryptic digests were first loaded to an Acclaim PepMap100 C18 guard column (3 μm, 100 Å, Dionex) at a flow rate of 3 μL/min for on-line desalting. Next, the separation of peptides was achieved using an Acclaim PepMap100 C18 capillary column (75 μm internal diameter × 150 mm, 2 μm, 100 Å, Dionex) at 0.35 μL/min in 120 min. The mobile phase A contained 2% ACN, 0.1% FA, and 97.9% water, whereas mobile phase B contained 0.1% FA in ACN. The LC gradient was as follows: solvent B was kept at 5% for the first 10 min, increased from 5 to 20% over 55 min, 20–30% over 25 min, 30–50% over 20 min, 50–80% over 1 min, kept at 80% for 4 min, decreased from 80 to 5% over 1 min, and finally maintained at 5% over 4 min.

The LTQ Orbitrap Velos was used in data-dependent acquisition mode. The scan events were set as a full MS scan of *m/z* 400–2,000 at a mass resolution of 15,000, followed by CID MS/MS scan repeated on the 10 most intense ions selected from the previous full MS scan with an isolation window of *m/z* 3.0. The normalized collision energy was set to 35% with an activation *Q*-value of 0.25 and activation time of 10 ms. The dynamic exclusion was enabled with repeat count of 2, repeat duration of 30 s, and exclusion duration of 90 s.

### LC-MS/MS Data Analysis

The raw data obtained from LC-MS/MS analysis were processed with the MaxQuant software version 1.6.3.4. Database search was performed against UniProtKB/Swiss-Prot mouse database. The search included cysteine carbamidomethylation as a fixed modification and variable modifications, including methionine oxidation and acetylation of protein N-terminal. Trypsin was specified as the proteolysis enzyme, and a maximum of two missed cleavages were allowed. For identification, the peptide precursor mass tolerance was 20 pm in the first search and 4.5 pm in the main search. As for fragments matching, a deviation of 0.5 Da was allowed. Only peptides with a minimum length of seven amino acids were considered for identification. The false discovery rate was set to be 0.01 at both peptide and protein levels. The minimum ratio count was set as two to determine the intensities of proteins for label-free quantification. Both unique and razor peptides were considered for quantification.

### Bioinformatics Analysis of the Proteomics Dataset

#### Data Preparation

The proteomics dataset, containing 2,902 protein identifiers, was preprocessed and normalized using Bioconductor’s DEP package in R environment (Zhang et al., 2018). A total of 187 identifiers were omitted prior to normalization, including 30 decoy database hits, 19 contaminants, 26 non-specific identifiers (mapped to multiple proteins), and 112 unmapped identifiers (uniport ID not mapped to any gene in Bioconductor’s Genome wide annotation for Mouse database). In addition, 1,126 proteins not present in all replicates of at least one condition were also removed (Supplementary Figures 1A–C). The filtered dataset, containing 1,589 protein IDs, was background corrected and normalized by variance stabilizing transformation (Supplementary Figures 1D,E). A heatmap of the presence status, for proteins with at least one missing value, showed a clustering pattern by condition (Supplementary Figure 2A), and their intensity distribution and cumulative fraction plots showed that missing values are enriched in proteins with low signal (Supplementary Figure 2B). This indicates that proteins are missing not at random, and therefore, MinProb, a left censored-imputation method, was applied (Supplementary Figure 2C). The final log2-transformed dataset is shown in Supplementary Table 1.

#### Fold-Change Analysis

Differential-expression analysis was done using protein-wise linear models combined with empirical Bayes statistics using DEP implementing the limma algorithm. For pairwise comparisons between groups, proteins with fold changes (FCs) beyond ± log2 (1.5) and Benjamini-Hochberg–adjusted *p*-values below 0.05 were considered significant.

#### Enrichment Analysis

Enrichment analysis on Gene Ontology (GO) biological process (BP), molecular function (MF), and cellular component (CC) terms was performed in R using topGO package^1^. GO terms were considered as significant when having a fold of enrichment greater than 2 with weighted-Fisher *p* < 0.05. Top enriched terms alongside corresponding proteins were visualized in Circos plots using circlize package in R environment (Gu et al., 2014). Enrichment analysis on Kyoto Encyclopedia of Genes and Genomes (KEGG) pathways was conducted in R environment using BioConductor’s clusterProfiler package (Yu et al., 2012).

#### Data Visualization

Principal component analysis (PCA) plots and heatmaps were drawn in R using Bioconductor’s DEP and ComplexHeatmap (Gu et al., 2016) Packages, respectively. Boxplots were drawn using ggplot2 R package^2^. The interaction network of all deregulated proteins and the network of enriched KEGG pathways were visualized in Cytoscape software (Shannon et al., 2003) using stringApp (Doncheva et al., 2019), and ClueGO (Bindea et al., 2009) applications, respectively.

#### WGCNA

Signed WGCNA with soft thresholding of 12 was implemented to construct a gene network using topological overlap dissimilarity measures. Identification of modules containing positively intercorrelated genes was done using dynamic tree cutting with deep slit relying on the shape of the branches in the dendrogram. Analysis was performed using the WGCNA package in R environment (Langfelder and Horvath, 2008). The gene–gene interaction network was visualized using Cytoscape software (Shannon et al., 2003).

## Results

### Pathways Contributing to Variance Among Conditions

The proteome of a mouse model of PD was investigated after intranigral transplantation of fetal DA neurons in a 6-OHDA mouse model of PD, using LC-MS/MS. Proteins were analyzed in five nigrostriatal-related brain regions (CPu, MFB, NAcc, Thal, and VM) at 1 day (SNLTD1 group) or 7 days (SNLTD7 group) after cell grafting, in comparison to sham-transplanted mice (Figures 1A,B). PCA was performed based on the expression level of all proteins in the dataset and showed that most of the variance between samples could be attributed to differences in tissue of origin (PC1), followed by time of transplantation (PC2) (Figure 1C). Indeed, this was confirmed when performing hierarchical clustering where samples from the same tissue were found to cluster together (Figure 1D). Besides, sham samples segregated apart from SNLTD1 and SNLTD7 groups in all five tissues, indicating higher variability between control and transplanted tissues than between days 1 and 7.

### Differentially Expressed Proteins, PCA, and Clustering

**FIGURE 2:**
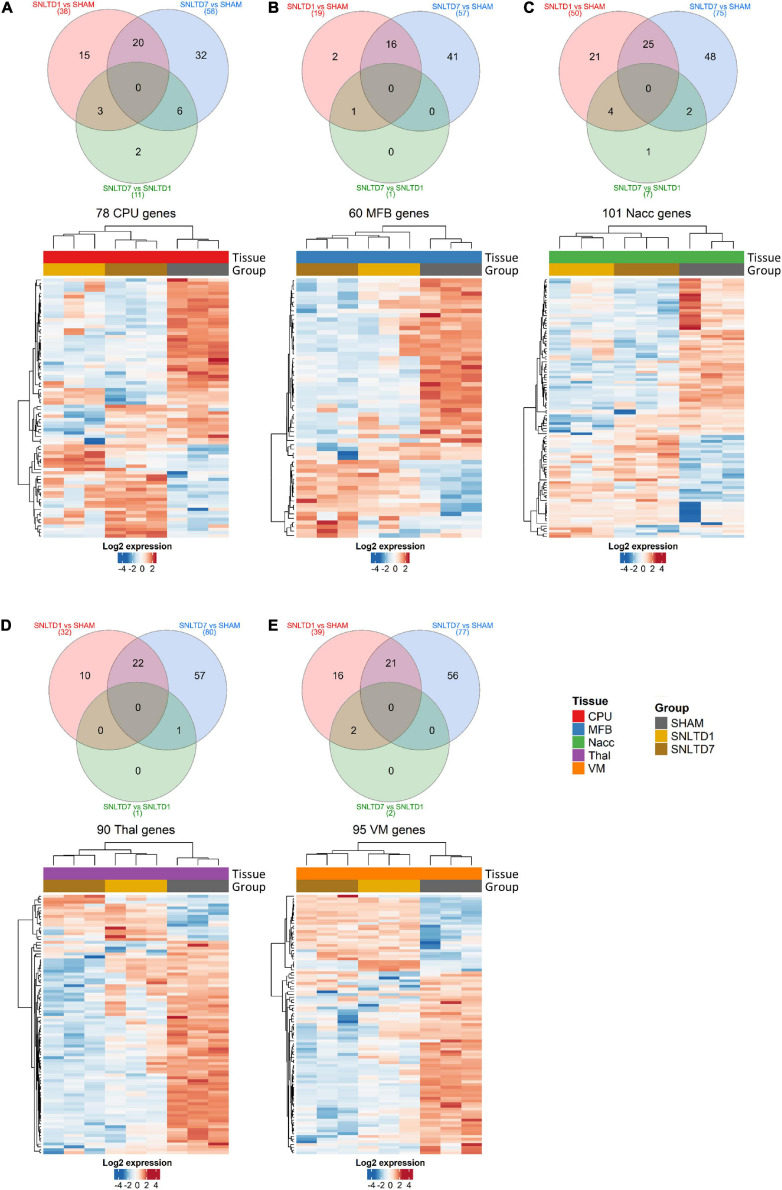
Differential-expression analysis. **(A–E)** Venn diagrams and heatmaps showing deregulated proteins in CPu, MFB, NAcc, Thal, and VM, respectively. In the heatmaps, red and blue colors represent high and low expression levels, respectively. Protein expression was centered and scaled prior to clustering. Hierarchical clustering was done using Euclidean distance with average linkage for samples, whereas Pearson correlation was used with average linkage for proteins.

Pairwise comparison was performed to identify differentially expressed proteins in each tissue at days 1 and 7 posttransplantation, each compared to sham, but also at day 7 compared to day 1 (Supplementary Table 2). Samples originating from CPu, MFB, NAcc, Thal, and VM tissues showed 2–21 proteins whose expressions were specifically altered at day 1 posttransplantation (Figures 2A–E, unique proteins in red Venn diagrams), whereas, on the other hand, a larger number of (32–57) were differentially expressed at day 7 posttransplantation compared to sham (Figures 2A–E, unique proteins in blue Venn diagrams). Interestingly, a large number of proteins deregulated at day 1 (50–84%) were also commonly altered at day 7 compared to sham, in all tissues (intersection of red and blue Venn diagrams). However, fewer proteins were deregulated at day 7 compared to day 1, with a total of 11, 1, 7, 1, and 2 proteins in CPu, MFB, NAcc, Thal, and VM, respectively (Figures 2A–E, green Venn diagrams). Among these, two proteins were specifically altered between days 7 and 1 in CPu, only one in NAcc and none in MFB, Thal, or VM. The identity and number of deregulated proteins within each region are shown in Supplementary Table 2.

Hierarchical clustering analysis showed that deregulated proteins were sufficient to distinguish between different time points in all tissues and that nearly 70% of deregulated proteins between days 1 and 7 after transplantation were down-regulated compared to sham (Figures 2A–E and Supplementary Table 2). Together, these data suggest a gradual, time-dependent proteomics changes posttransplantation and indicate that, on average, approximately 25% of deregulated proteins are shared between days 1 and 7, as compared to sham. However, the protein expression profiles at days 1 and 7 were very similar confirming the observations in Figures 1C,D.

### Common and Tissue-Specific Proteins, Enrichment Analysis, and GO

**FIGURE 3:**
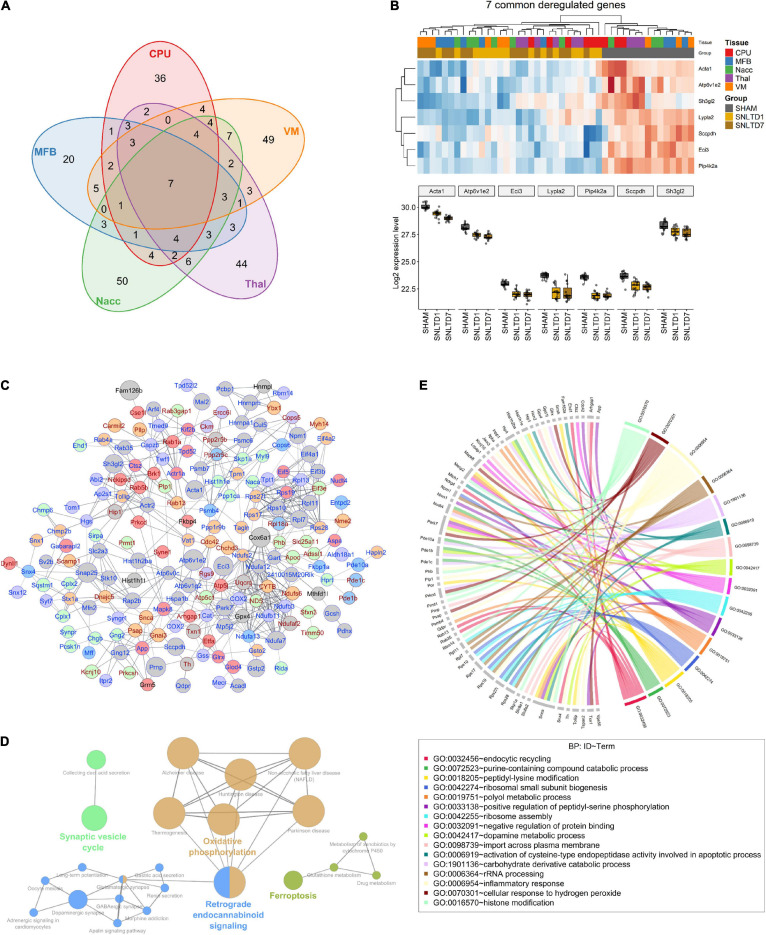
Commonly deregulated proteins across tissues. **(A)** Venn diagram resuming the intersections between deregulated proteins (both up- and dow-regulated) in CPu, MFB, NAcc, Thal, and VM. **(B)** Heatmap (upper) and boxplot (lower) showing the expression profile of the seven commonly deregulated proteins in all tissues. **(C)** String-db interaction network of 183 deregulated proteins. Nodes colored in red, blue, green, purple, and orange represent CPu, MFB, NAcc, Thal, and VM-specific proteins, respectively. Gray nodes represent proteins deregulated in more than one tissue. Node size is proportional to the number of tissues in which the correspondent protein is deregulated. Symbols for proteins that are consistently up-regulated at days 1 and 7, compared to sham, are annotated in red, whereas those for consistently down-regulated proteins are annotated in blue. Edge width is proportional to interaction score according to string-db. Only interactions with a score above 0.6 were shown. **(D)** Network of KEGG pathways that are enriched in commonly deregulated genes. Nodes representing interconnected pathways are filled with the same color. **(E)** Circos plot showing Gene Ontology (GO) for biological processes (BP) that were significantly deregulated in the dataset.

To better understand the patterns of proteomic changes between tissues, we examined the overlaps between altered proteins in all tissues. Analysis of all deregulated proteins showed that 199 proteins (72%) are tissue-specific, whereas only seven proteins are commonly altered among all tissues (Figure 3A). The latter proteins included Acta1, Atp6v1e1, Eci3, Lypla2, Pip4k2a, Sccpdh, and Sh3gl2, which were down-regulated in all tissues at both days 1 and 7 (Figure 3B). Interestingly, GO enrichment analysis revealed that four of these seven proteins (Eci3, Lypla2, Pip4k2a, and Sccpdh) are involved in cellular lipid metabolic processes, with 8.5-fold enrichment (Supplementary Table 3).

Next, we generated a protein–protein interaction (PPI) network using String-db, which revealed that 66% of the deregulated proteins are functionally correlated (Figure 3C). Notably, among proteins altered in more than one tissue (gray nodes), a majority was consistently down-regulated at both days 1 and 7 posttransplantation, compared to sham (protein symbols highlighted in blue).

Globally, KEGG enrichment analysis of the 277 deregulated proteins revealed that they are involved in PD, oxidative phosphorylation, synaptic vesicle cycle, dopaminergic synapse, and ferroptosis, among other pathways (Figure 3D). A closer look at PD proteins (*n* = 18) showed high variability in their deregulation pattern between tissues (Supplementary Figure 3). For instance, Cox6a1 increased after transplantation in Thal and CPU; however, it decreased in VM and MFB. On the other hand, at day 7, Ndufa7 was significantly down-regulated in all tissues except MFB. Besides, analysis of GO BPs showed that these proteins are enriched in oxidative stress, inflammatory response, and apoptotic and DA metabolic processes (Figure 3E). However, tissue-wise GO enrichment analysis of BP, MF, and CC terms showed that each tissue has a distinct set of up- and down-regulated pathways (Supplementary Table 4). Interestingly, further examination of these pathways revealed that VM, Thal, and NAcc are characterized by the down-regulation of proteins involved in glutathione metabolic process (Supplementary Figure 4). This suggests the existence of potential oxidative stress in these tissues posttransplantation.

**FIGURE 4:**
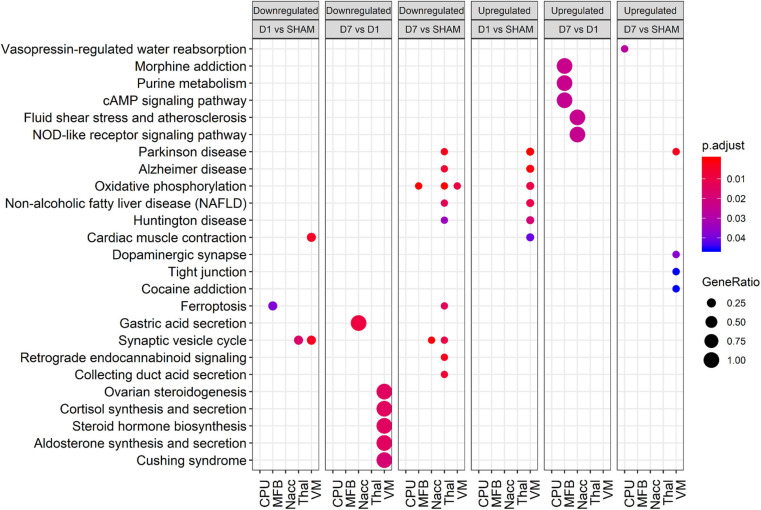
Bubble plot showing enriched KEGG pathways for various comparisons in each tissue. Rows and columns represent KEGG terms and comparisons, respectively. Bubble size and color reflect gene ratio and significance. Only terms with adjusted *p* < 0.05 in at least one condition are shown. Only the top 10 significant terms of each group were plotted.

In accordance with the tissue-wise GO enrichment analysis, study of deregulated KEGG pathways revealed that these are indeed not equally altered across all tissues (Figure 4). For instance, whereas oxidative phosphorylation was down-regulated in MFB, Thal, and VM at day 7 posttransplantation, it was not affected in either CPu or NAcc. On the other hand, VM was the most enriched tissue for up-regulated pathways both at days 1 and 7 posttransplantation. Compared with sham, only VM showed enriched pathways at day 1 posttransplantation, including pathways involved in oxidative phosphorylation and Central Nervous System (CNS) diseases, whereas at day 7 posttransplantation, proteins involved in dopaminergic synapse were up-regulated only in VM. In day 7 vs. day 1 posttransplantation, there was an up-regulation in proteins involved in neurotransmitter signaling in MFB, whereas those involved in NOD-like signaling were up-regulated in NAcc.

In summary, tissues showed high variability in their enrichment patterns posttransplantation, with few common pathways.

### Network Analysis and Identification of Hub Proteins

**FIGURE 5:**
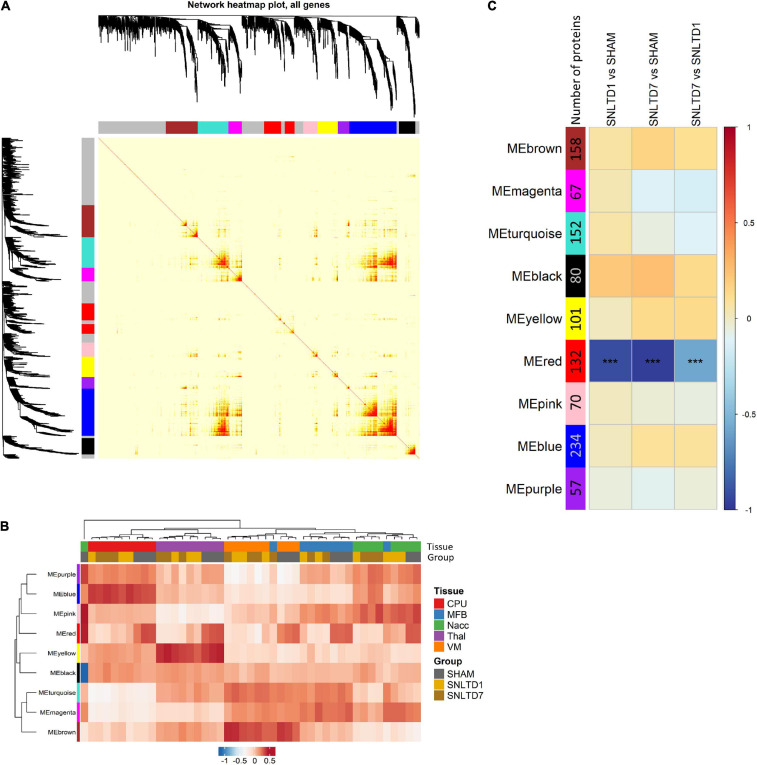
Weighted gene coexpression network analysis (WGCNA). **(A)** Topology overlap matrix showing nine modules represented by different colors. The gray color represents 538 background proteins that were not attributed to any of the nine modules. **(B)** A heatmap of module eigengenes (MEs) summarizing the various identified modules. ME, calculated for each module, is a synthetic gene defined as the first principal component (PC1 from PCA) of the expression matrix of the corresponding module. **(C)** A module–trait relationship plot showing Pearson correlation between the MEs from various modules and the following binary comparisons: SNLTD1 vs. SHAM, SNLTD7 vs. SHAM or SNLTD7 vs. SNLTD1, regardless of tissue of origin.

We applied WGCNA to better understand transplantation-induced proteomic changes across tissues. Unlike FC analysis that is limited to top deregulated proteins, WGCNA uses the whole dataset to identify modules of highly interconnected proteins. Nine distinct modules were generated, each represented by a specific color (Figure 5A), ranging in size from 234 proteins (blue module) to 57 proteins (purple module). Each module was then represented by an eigengene, a synthetic gene, which is the dimension that explains the highest percentage of variance (PC1) from PCA. Hierarchical clustering of module eigengenes (MEs) revealed that many modules were correlated with specific tissues (Figure 5B). For instance, the blue, yellow, and brown MEs were highly expressed, respectively, in CPu, Thal, and VM, whereas these same tissues weakly expressed the turquoise, pink, and purple MEs, respectively. This is consistent with the fact that most variability in the dataset comes from differences between tissues (Figures 1C,D). Module–trait correlation analysis revealed that, among the nine identified modules, the ME of the red module was negatively correlated with days 1 and 7 compared to sham, but also day 7 compared to day 1 (Figure 5C).

**FIGURE 6:**
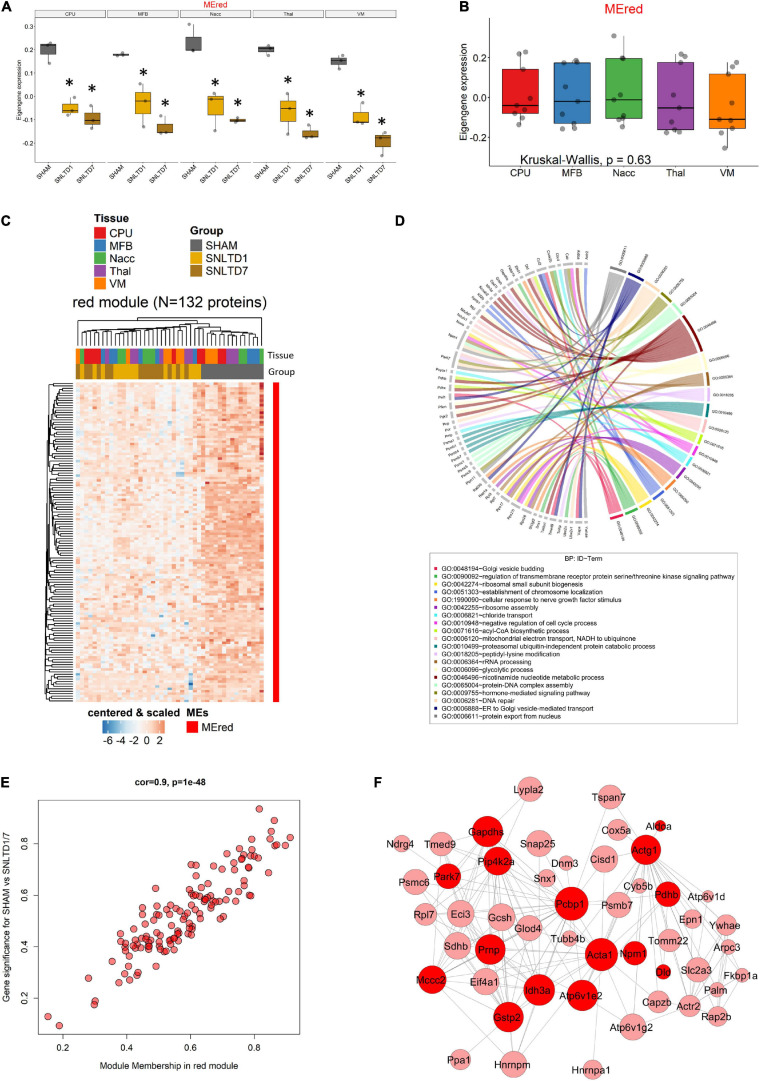
Analysis of the red module. **(A)** Boxplot showing the expression of the red module’s ME (MEred) across sham, day 1, and day 7 conditions for each tissue. *Corresponds to statistical significance compared to Sham. **(B)** Boxplot illustrating the expression of MEred across tissues. Kruskal–Wallis test was used for multiple comparisons. **(C)** Heatmap showing the expression profile of the 132 proteins forming the red module. **(D)** Circos plot displaying GO biological process (BP) terms that are significant enriched in red module proteins. **(E)** Module membership (MM) was defined as the correlation between gene expression and module eigengene. Gene significance (GS) was defined as the correlation between gene expression and SHAM compared to the combined days 1 and 7 conditions, regardless of regions. **(F)** Protein–protein interaction network of hub genes in the red module. Node size and edge width are, respectively, proportional to module membership (MM) and weighted correlation. Only genes with weighted correlation greater than 0.08 are shown. Proteins related to metabolic processes are highlighted in bright red color.

This suggests that the global expression of red module proteins is high in sham but decreases with time after engraftment, as confirmed in Figure 6A. Interestingly, the red ME showed no difference between CPu, MFB, NAcc, Thal, and VM tissues (Figure 6B). As expected, unsupervised hierarchical clustering revealed that the 132 proteins in the red module displayed lower expression at days 1 and 7 in all tissues, compared to sham samples which clustered together (Figure 6C). Unsurprisingly, this module shared 62 proteins that were down-regulated posttransplantation in the global analysis (Supplementary Figure 5), including all seven common proteins described in Figure 3B. GO analysis revealed that red module proteins are mostly enriched in proteins related to nicotinamide nucleotide metabolism and are involved in the regulation of serine/threonine kinase receptor singling and cellular response to nerve growth factor stimulus (including Ehd1, Sh3gl2, Rab35, Rap1a) (Figure 6D and Supplementary Table 5). Strikingly, the 132 proteins in the red module showed strong positive correlation (*r* = 0.9, *p* = 10^–48)^) between their module membership and their higher expression in sham, compared to transplantation at day 1 or day 7 (Figure 6E and Supplementary Table 6). PPI network analysis, using weighted-correlation data, revealed a set of 48 highly interconnected hub proteins within the red module (Figure 6F). It is important to note that seven metabolic proteins (bright red nodes) were central in this network and especially involved in mitochondrial function, such as Idh3a, Pdhb, and Sdhb.

**FIGURE 7:**
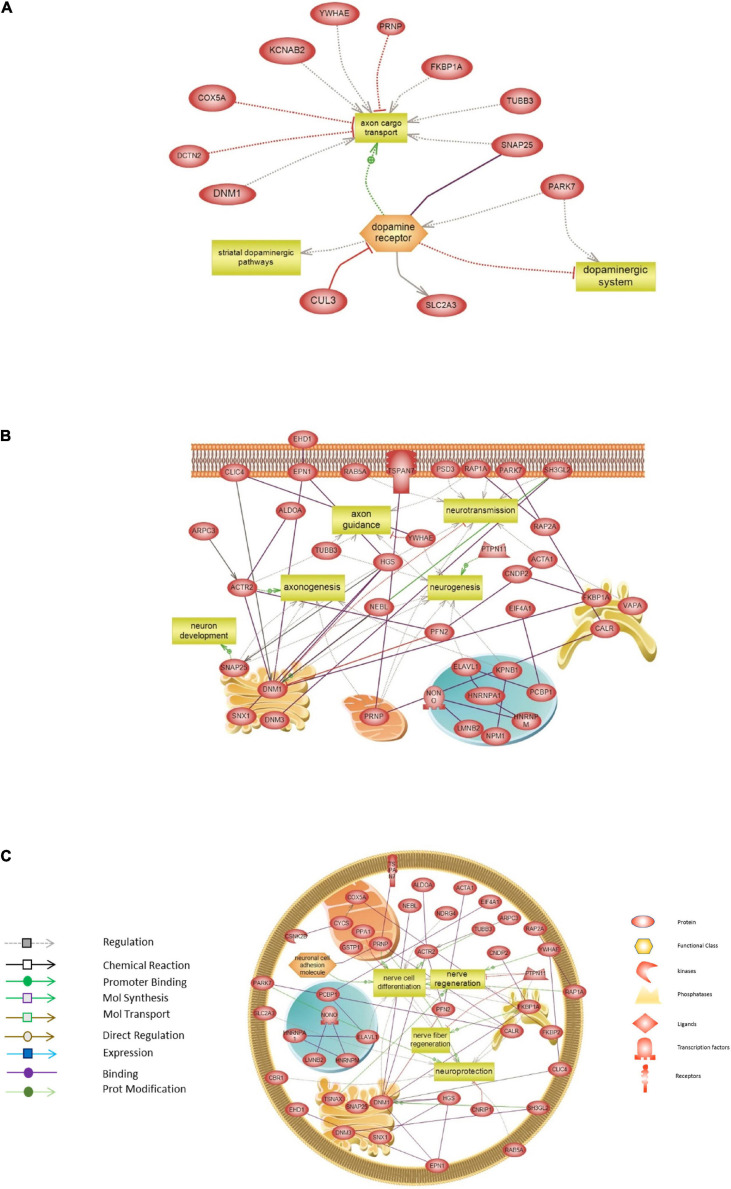
Targeted molecular and biological pathways’ interaction map analysis performed on the genes from the red module. Using Pathway Studio 10.0, altered differential proteins relevant to transplantation in mouse brain were analyzed. Direct interactions algorithm was used to generate and map the targeted network of biological processes and interactions among altered proteins in: **(A)** The dopaminergic system. **(B)** Neurogenesis, axonogenesis, neurotransmission, and neuron development. **(C)** Neuroprotection, nerve cell differentiation, and regeneration. Each of the proteins is displayed within a subcellular compartment or organelle. The shape of each given protein is indicative of its functional class as shown in the legend. Included in the legend is the definition of the lines connecting two proteins.

Finally, targeted molecular and biological pathways’ interaction map analysis was performed on the genes from the red module, using Pathway Studio and Ingenuity Pathway analysis (Figure 7). Altered differential proteins relevant to transplantation were mapped on the targeted network of BPs and interactions in the DA system (Figure 7A), neurogenesis, axonogenesis, neurotransmission and neuron development (Figure 7B), and neuroprotection, nerve cell differentiation, and regeneration (Figure 7C). More precisely, interaction pathway network analysis revealed several proteins implicated in mediating axonal transport. Among these proteins are FKBPA1, DNM1, PRNP, DCTN2, and COX5, which are known to be involved in pathways devoted to disrupting the axon cargo transport. In addition, DA receptors were devoted to regulating striatal dopaminergic pathways and altering the dopaminergic system, which was also modulated by PARK7 (Figure 7A). The proteins and corresponding BPs identified are summarized in Supplementary Table 7. Other network interactions revealed several proteins to be connected to neurotransmission, neurogenesis, axonogenesis (DNM1, PRNP, SNAP25), axon guidance (ACTR2, TUBB3, YWHA), and neuron development (SNAP25) (Figure 7B and Supplementary Table 7). In addition, several direct interactions were also revealed among the different proteins of the red module modulating the BPs (Figure 7B). In this regard, DNM1 directly interacts with SH3GL2, which was down-regulated after transplantation in all tissues. The cell surface glycoprotein TSPAN7 was found to directly interact with the prion protein PRNP located in the mitochondria. Besides, the proteome network highlighted proteins involved in nerve cell differentiation (PRNP, FKBP1A, DNM1, TUBB3), nerve and nerve fiber regeneration (FKBP1A, PTPN11), and neuroprotection (PRNP, PARK7, FKBP1A) (Figure 7C). These pathways and the relevant proteins are presented in Supplementary Table 7.

## Discussion

In this study, we aimed to identify differentially expressed proteins in five regions related to the reconstruction of the nigrostriatal pathway after intranigral transplantation of fetal VM cells in a mouse model of PD (Gaillard et al., 2009; Thompson et al., 2009). To achieve this, we used non-targeted proteomic analysis based on mass spectrometry. Among top cellular pathways involved in tissue-specific response to transplantation, we identified pathways related to lipid, mitochondrial and DA metabolism, protein translation, and nerve growth. Interestingly, at 7 days posttransplantation, when grafted cells reach their striatal final target, a high number of proteins (63%) showed modulation of their expression in all five regions in comparison to non-grafted sham condition.

Moreover, our results showed that 72% of the deregulated proteins were tissue-specific, in line with the various functions and cellular compositions of these five brain regions. Indeed, VM, MFB, and CPu constitute the DA nigrostriatal pathway that is restored after grafting, as shown by Gaillard et al. (2009). VM contains the somas of grafted fetal neurons, and the MFB constituted of axons and projections of grafted neurons, whereas CPu holds the axonal terminals and projections of grafted neurons. Thal and NAcc are neighboring regions of the nigrostriatal pathway that express axon guidance cues allowing the ventral trajectory of the nigrostriatal pathway that is established during development (Deschamps et al., 2009; Kalaani et al., 2016).

Differential-expression analysis showed that 277 proteins were deregulated after transplantation, in which seven proteins (Acta1, Atp6v1e1, Eci3, Lypla2, Pip4k2a, Sccpdh, and Sh3gl2) were especially down-regulated after grafting. This decrease of expression was progressive from days 1 to 7 after transplantation across all regions, in comparison to non-grafted animals. These proteins are important for the formation of lipids and recycling of DA vesicle at the synapse. Moreover, it is known that 6-OHDA treatment of SH-SY5Y cells, a well-established *in vitro* model of PD, deregulates multiple lipid classes, including phosphatidylcholine (PC), phosphatidylglycerol, phosphatidylinositol, phosphatidylserine, sphingomyelin, and total cholesterol (Xicoy et al., 2020). Knowing that patients with neurodegenerative conditions such as PD are frequently characterized by dysregulated lipid metabolism, which in turn affects the structure of lipid rafts (Grassi et al., 2019), it would be interesting to further study these modified lipid rafts to explore a possible restoration of lipid metabolism after intranigral transplantation of fetal DA neurons in comparison to sham condition. Among these deregulated proteins, the actin α1 skeletal muscle (Acta1), which is implicated in cell motility, structure, and integrity, and Atp6v1e2, an ATPase H^+^ transporter essential for catabolic processes, are both known to be prognosis markers in gliomas as they are involved in invasion and motility of cancer cells (Yang et al., 2012; Ohtaki et al., 2017). Moreover, Pip4k2a expression was down-regulated between days 1 and 7 posttransplantation. This protein is known to be involved in the biosynthesis of phosphatidylinositol-4, 5-bisphosphate, which regulates exocytotic fusion of synaptic vesicles (glutamate and DA) with the plasma membrane. Polymorphisms in this gene are observed in schizophrenia (Kaur et al., 2014), and our results suggest that this gene could also be linked to PD. Moreover, the peroxisomal isomerase Eci3 and the acyl-protein thioesterase 2 (Lypla2), a fatty acid hydrolase crucial for lipid degradation, were both found to be modulated in our model after intranigral transplantation of fetal DA neurons. Overall, our results suggest that these proteins known to be involved in lipid metabolism and deregulated in PD may also play a role in the functional reconstruction of the nigrostriatal pathway described after intranigral transplantation of fetal DA neurons (Gaillard et al., 2009). Moreover and interestingly, these proteins could also influence synuclein metabolism as it has been shown that synuclein accumulation and Lewy bodies formation may be linked to peroxisome dysfunction (Islinger et al., 2018). Indeed, Pex2^–/–^, Pex5^–/–^, and Pex13^–/–^ mouse models of disrupted peroxisome biogenesis exhibit increased α-synuclein phosphorylation, oligomerization, and inclusion body formation (Yakunin et al., 2010).

Analysis of GO BPs showed that the 277 deregulated proteins are mainly involved in inflammatory response and apoptotic and DA metabolic processes, which are known to be related to PD pathogenesis. Indeed, the 6-OHDA mouse model of PD is known to induce oxidative stress and inflammation as well as a shutdown of DA metabolism (Bove and Perier, 2012). It has been revealed that treatment by 6-OHDA induces DA neurons death triggering oxidative stress, with lipid peroxidation, production of hydrogen peroxide, and inhibition of complex mitochondrial respiratory chain as well as membrane potential collapse (Ogawa et al., 1994; Gomez-Lazaro et al., 2008). Here, we showed that 7 days after transplantation, the expression of oxidative stress–related proteins decreases compared to sham animals. For instance, expression of protein deglycase DJ-1, encoded by park7 and highlighted in the PPI network of hub proteins in the red module, decreases after grafting. As this protein acts as a sensor for oxidative stress to protect neurons from cell death (Oh et al., 2018), this suggests that oxidative reaction due to the 6-OHDA lesion decreases along with the presence of the grafted cells. Nevertheless, proteins involved in oxidative phosphorylation pathway (leading to ATP production) are down-regulated 7 days after transplantation, especially in the SN, where the somas of grafted cells are located, suggesting that mitochondrial function is not yet totally restored at this time point. It would be then interesting to test whether a potential restoration of protein expression to basal level would happen later than 7 days, that is, few weeks after transplantation, as it has been carried out in other therapeutic approaches in animal models of PD (Teixeira et al., 2017; Mendes-Pinheiro et al., 2018).

In contrast, in the VM and CPu, GO terms were mostly up-regulated 7 days after grafting compared to sham. In the VM, containing the somas of grafted neurons, up-regulated proteins were involved in BPs, such as regulation of autophagy and phospholipid metabolism, and CCs, such as secretory granule membranes, clathrin-coated vesicle, synaptic vesicle, recycling endosome, and cytoplasmic vesicle membrane. Interestingly, up-regulation of these proteins, especially α-synuclein (Snc-α) and tyrosine hydroxylase (Th), suggests that the machinery of neurotransmitter, and particularly DA release, is restored in grafted cells 7 days after transplantation. Indeed, 6-OHDA lesion in MFB is known to induce a decrease of tyrosine hydroxylase expression in the CPu at 3, 7, and 14 days after the lesion (Fuller et al., 2014). Our results demonstrated that transplantation restores the level of tyrosine hydroxylase expression, suggesting a possible functional repair of the nigrostriatal pathway. Moreover, CCs of lamellipodium and growth cones were up-regulated 7 days after transplantation, whereas, surprisingly, the motility machinery related to actin cytoskeleton and extracellular matrix molecules decreased. This apparent discrepancy may be explained by the fact that the majority of the axons-derived DA neurons have reached their terminal targets at 7 days postgrafting, and hence, their overall motility is diminished; however, the connectivity is still refined at the growth cone.

In the CPu, which is the main target region of axons of the grafted neurons, expression of mitochondrial proteins and cytoplasmic vesicle membrane-related proteins was up-regulated 7 days after grafting, suggesting a restoration of the mitochondrial function that is a signature of oxidative stress attenuation. Taken together, these results are in line with a functional repair observed in this model of cell therapy in PD (Gaillard et al., 2009) and provide some indications about the mechanisms involved in the nigrostriatal reconstruction process.

Surprisingly, the expression of proteins related to the regulation of serine/threonine kinase receptor signaling and to cellular response to nerve growth factor stimulus (including Ehd1, Sh3gl2, Rab35, and Rap1) were progressively down-regulated after grafting. This suggests that in response to 6-OHDA lesion of SN DA neurons, the surrounding tissue may produce neurotrophic factors to allow the growth of grafted axons to their striatal targets. After transplantation, this production then gradually decreases, especially in the nigrostriatal pathway (CPu, MFB, and VM), until grafted axonal terminals have reached their striatal targets 7 days after grafting. Among these proteins, endophilinA, encoded by Sh3gl2, is required for synaptic vesicle endocytosis and BDNF-dependent neurite outgrowth. The decrease of expression of endophilinA, despite the presence of grafted cells 7 days after transplantation, suggests synaptic vesicle trafficking defects, as described previously (Xiong et al., 2014). It is worth mentioning that DJ-1, encoded by PARK7, also plays a critical role in synaptic vesicle trafficking and that its deficiency leads to impaired synaptic vesicle endocytosis (Kyung et al., 2018). As expression of DJ-1 also decreased after grafting, it may also contribute to defects in vesicle trafficking along with endophilinA. However, these defects may not be sufficient to alter grafted DA neuron function or may be compensated, as we previously observed an anatomic and functional reconstruction of the nigrostriatal pathway in this model (Gaillard et al., 2009). BDNF is known to enhance the survival of DA neurons and to improve DA neurotransmission and motor performance (Palasz et al., 2020). Thus, the action of BDNF could occur early after lesion, in order to promote the survival of DA grafted neurons and then decrease after transplantation, concomitant with the navigation of grafted cells to their final target. Moreover, a recent study on PD patients has identified rare variants in SH3GL2, which may contribute to PD risk (Germer et al., 2019). Indeed, SH3GL2 variants implicate defective synaptic vesicle endocytosis that may contribute to the degeneration of midbrain DA neurons in patients (Nguyen et al., 2019). These results are in line with the importance of endophilinA in the survival and protection of DA neurons. Further studies would be beneficial to determine the implication of this protein in the reconstruction of the nigrostriatal pathway after cell therapy. If endophilinA is identified as crucial for grafted neuron survival, it may constitute a molecular tool to ensure neuroprotection and to ameliorate the transplantation efficiency.

Finally, Tspan7 was found as one of the hub proteins in the red module, although not captured among the commonly deregulated proteins. In fact, Tspan7 expression decreased with the growth of grafted DA fibers toward their targets, although it mediates signal transduction events that play a role in the regulation of cell development, activation, growth, and motility. This protein is a cell surface glycoprotein that combines with integrins and that play a role in the control of neurite outgrowth (Usardi et al., 2017). Integrins are known to be linked to extracellular matrix proteins and axonal guidance molecules to promote axonal growth and navigation. For example, Semaphorin7A axon guidance molecule promotes dendrite growth, complexity, and spine development through β1-subunit–containing integrin receptors in the adult hippocampal dentate gyrus (Jongbloets et al., 2017). However, a new role for TSPAN7 has been described recently as a regulator of D2 DA receptors (Lee et al., 2017). The authors showed that TSPAN7 is associated with D2 DA receptors and reduced their plasma membrane expression by enhancing the internalization process. TSPAN7, which resides in the membrane of early and late endosomes, was also shown to promote the internalization of D2 DA receptors and their location to endosomal compartments. Furthermore, TSPAN7 deficiency increases surface location of D2 DA receptors concomitantly with a decrease of its endocytosis. Finally, TSPAN7 negatively affects D2 DA receptor–mediated signaling. Taken together, the decrease of TSPAN7 expression observed after transplantation, associated with endophilin A1 down-regulation, suggests that, up to 7 days after transplantation, these genes may be shut down to promote DA transmission through a decrease of DA and D2 DA receptors endocytosis.

On another aspect, TSPAN7 was shown to interact directly with PRNP, one of the proteins in the red module that was deregulated after transplantation in our model. Pathway analysis revealed that PRNP mediates axonal transport, neuroprotection, and neurotransmission. Indeed, there is increasing evidence that PrP^*C*^, a cellular glycoprotein encoded by PRNP gene, plays important roles in neuroprotection (Linden, 2017). However, aggregates of misfolded PrP^*C*^ were reported to block axonal transport, intervene in neurotransmission, or induce apoptotic pathways (Cecarini et al., 2010; Halliday et al., 2014). PrPc was further proposed to regulate striatal dopaminergic transmission through alterations of DA receptors (Rial et al., 2014). Particularly, genetic deletion of PrPc in Prnp^–/–^ mice resulted in significant reduction of D1 (but not D2) DA receptors in the striatum. This indicated that PrPc may interfere in neurodegenerative brain disorders associated with DA, namely, in PD (Rial et al., 2014). Indeed, PRNP may be an interesting target in PD, especially that PrP^C^ has been shown to interact with α-synuclein and may be involved in early stages of the disease (Ferreira et al., 2017). Therefore, it is tempting to speculate that in our study PRNP along with Tspan7 may contribute to DA neurotransmission after transplantation by acting on DA receptors. Moreover, FKBP1A is among the red module proteins regulating the serine/threonine kinase receptor signaling. Pathway network analysis revealed that FKBP1A is associated with several biological pathways including neuroprotection, nerve regeneration, and axonal transport. As demonstrated in previous studies, alterations in FKBP1A may lead to impairment in axonal transport and defects in target proteins assembly (Sugata et al., 2009). Interestingly, FKBP1A protein level was augmented in a 6-OHDA model of PD, as well as in brains of PD patients (reviewed by Chattopadhaya et al., 2011), and it has been shown to play a key role in α-synuclein toxicity in a rat model of PD (Caraveo et al., 2017). Protein network analysis revealed the interaction of dynamin 1 protein (DNM1) with several other proteins including FKBP1A and SH3GL2. Notably, DNM1 plays a key role in regulating synaptic vesicle endocytosis, as well as neurotransmission (Patterson et al., 2008), and forms a complex with endophilinA (encoded by SH3GL2), which regulates clathrin-mediated endocytosis (Sundborger et al., 2011). As SH3GL2 and FKBP12 may be potential effectors in PD; therefore, investigating DNM1-associated pathways could provide new insights to target the impairments in synaptic transmission in transplantation therapeutic interventions. Furthermore, one of the proteins associated with modulating several biological pathways including neurotransmission is synaptosomal-associated protein (SNAP25), which was identified in the red module. It was previously shown that SNAP25 is essential for proper neurotransmission (Wei et al., 2013). Moreover, mutations of SNAP25 in mice were previously reported to disrupt DA neural transmission in the dorsal striatum, which leads to the possibility that SNAP25 may play a major part in synaptic DA release (Davids et al., 2003; Yang et al., 2019). Recent evidence stated that in the cerebrospinal fluid of patients with PD, SNAP25 levels were elevated and contributed to the severity of motor and cognitive symptoms (Bereczki et al., 2017). Besides, polymorphisms in SNAP25 gene, which were suggested to play a role in pathogenesis of PD, may provide protection against the onset of the disease (Agliardi et al., 2019). As a whole, SNAP25 may confer a potential candidate in early therapeutic approaches targeting the early pathological complications in PD occurring at the synaptic sites.

Concerning the relevance of PD model used in this study, the efficacy of 6-OHDA has been shown to be very neurotoxic, affecting more than 90% of the dopaminergic neurons located in the SN (Torres et al., 2011). In addition, the degeneration of fibers in the striatum has been recently shown to be fully established within 1 week following 6-OHDA administration; however, the loss of neurons continues to progress over time, becoming fully established 3 weeks after the 6-OHDA injection (Rentsch et al., 2019). Moreover, our laboratory has already shown, using immunohistochemistry, that intranigral injection of 6-OHDA induces significant degeneration, between 80 and 95% loss of dopaminergic neurons, concomitant with motor deficits. Furthermore, intranigral transplantation of dopaminergic neurons was revealed to induce significant motor recovery (Gaillard et al., 2009; Gaillard and Jaber, 2011). However, one limitation to this study arises from the inability to include a behavioral assessment of the PD model, such as the rotacount, based on the experimental timeline used here. Concerning the motor behavior, the relationship between 6-OHDA lesion and turning after amphetamines is complex. Schwarting and Huston (1996) established that injection of amphetamine elicited ipsiversive turning in animals with massive lesions of >95% DA depletions, 4 days after lesion. Although preceded by an initial period of contraversive turns up to day 7, this initial contraversive asymmetry diminished from days 5 to 7, and the number of wider turns increased. On the other hand, only ipsiversive turns appeared after day 7. In fact, an amphetamine-challenge can elicit contraversive turning within the first days after lesion, and this response can be attributed to the release of otherwise non-functional DA pools in the damaged hemisphere, especially the neostriatum. Thereafter, such pools are presumably lost in the damaged hemisphere, and the direction of turning is attributed mainly to the release of DA in the intact hemisphere. Therefore, it is not reliable to use motor test before 2 or 3 weeks after lesion. This corroborates the more recent observation that the motor effects of the lesion are measurable only 2 or 3 weeks after lesion (Heuer et al., 2012). Taken together, these observations demonstrate that the dopaminergic fiber degeneration is effective 7 days after the lesion; however, it would not be reliably measurable with a motor test such as rotacount evaluation.

Taken together, identification of biological networks among the altered differential proteins relevant to transplantation shows multiple interaction networks associated with processes such as neuroprotection, neurogenesis, neurotransmission, and axonal transport, which may provide novel targets in therapeutic approaches of PD. In the future, and in order to identify the precise impact of these deregulated proteins on the nigrostriatal pathway reconstruction, it would be crucial to test the effects of loss and gain of function of these proteins after grafting. These proteins may constitute valuable therapeutic targets to ameliorate cell therapy efficiency in PD patients.

## Data Availability Statement

The original contributions presented in the study are publicly available. Data are available via ProteomeXchange with identifier PXD022193.

## Ethics Statement

The animal study was reviewed and approved by the Ethics Committee of the University of Poitiers.

## Author Contributions

SM, AG, and LP performed the mouse model and collected samples. FK, MZ-M, PM, and YM performed proteomics experiments. HD, AN, and KZ performed proteomics analysis, figures, tables, and supplementary figures and tables. HD, LP, and KZ wrote the manuscript. AG, LP, and KZ supervised the work. All authors revised and corrected the final manuscript of the manuscript.

## Conflict of Interest

The authors declare that the research was conducted in the absence of any commercial or financial relationships that could be construed as a potential conflict of interest.

## Footnotes

1 https://bioconductor.org/packages/release/bioc/html/topGO.html

2 https://cran.r-project.org/web/packages/ggplot2/
