# Supplementary figures and images for: Analysis of the Neuroproteome Associated With Cell Therapy After Intranigral Grafting in a Mouse Model of Parkinson Disease
Source: Front Neurosci. 2021 Mar 11;15:621121. doi: 10.3389/fnins.2021.621121 (PMC7991918; doi:10.3389/fnins.2021.621121)

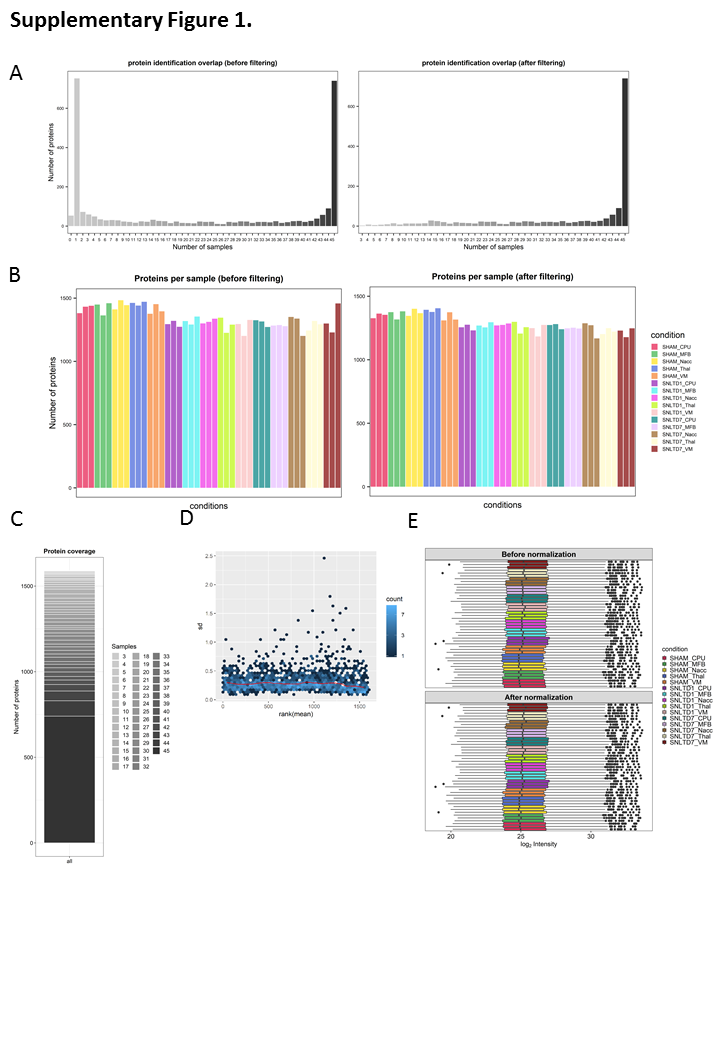

Supplement: Supplementary file 8 [file Image_1.TIF]

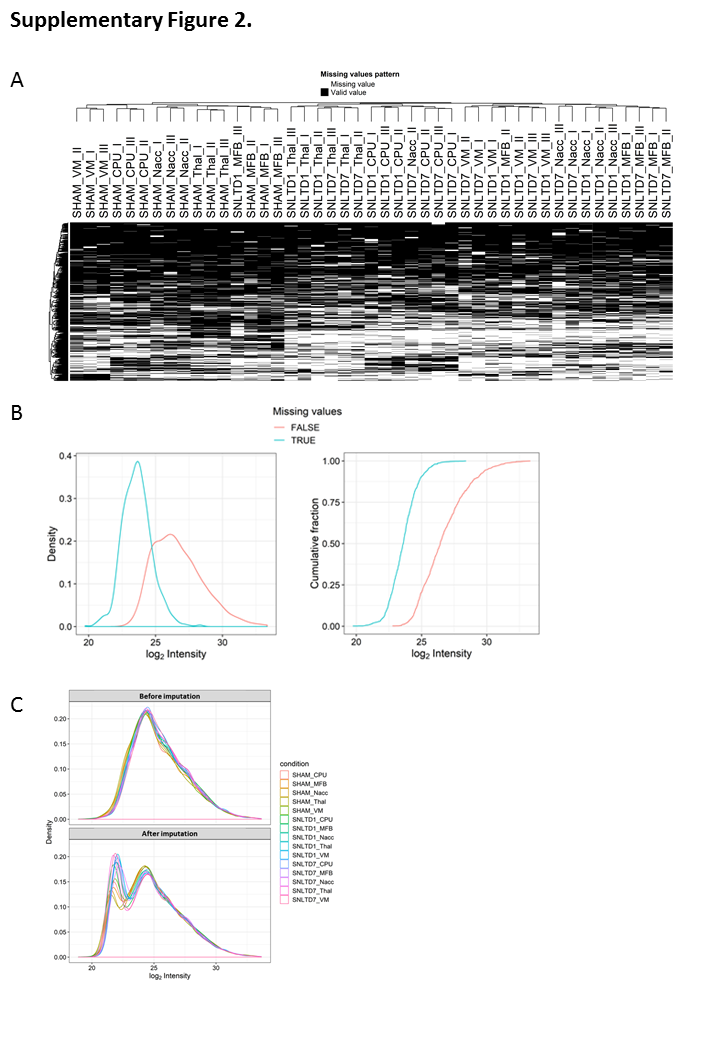

Supplement: Supplementary file 9 [file Image_2.TIF]

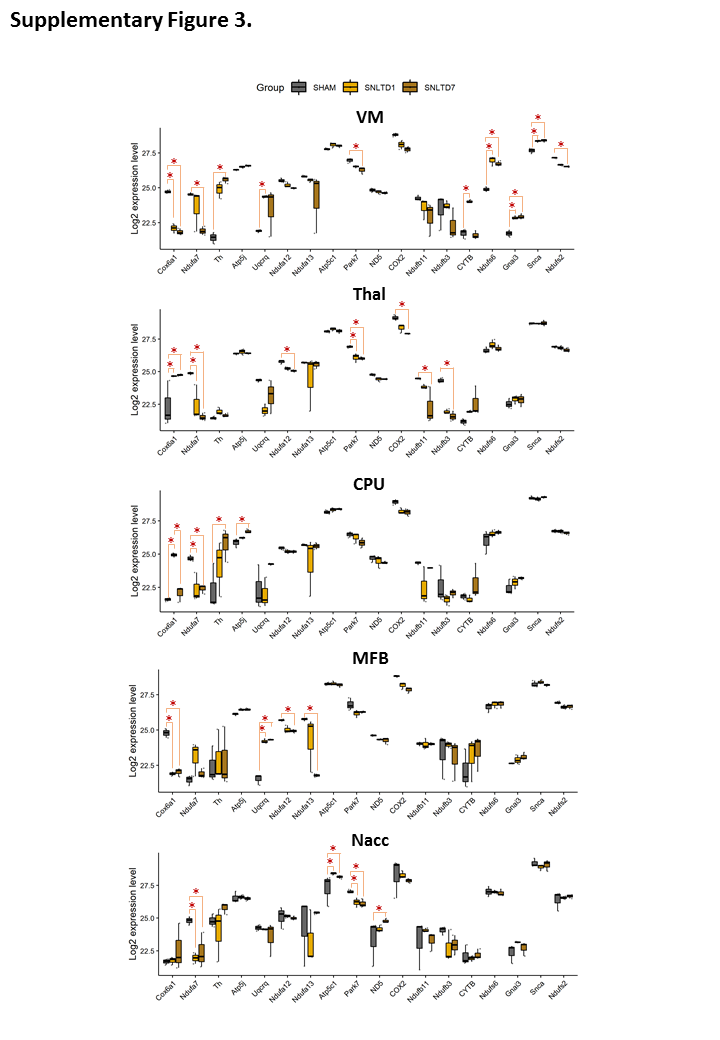

Supplement: Supplementary file 10 [file Image_3.TIF]

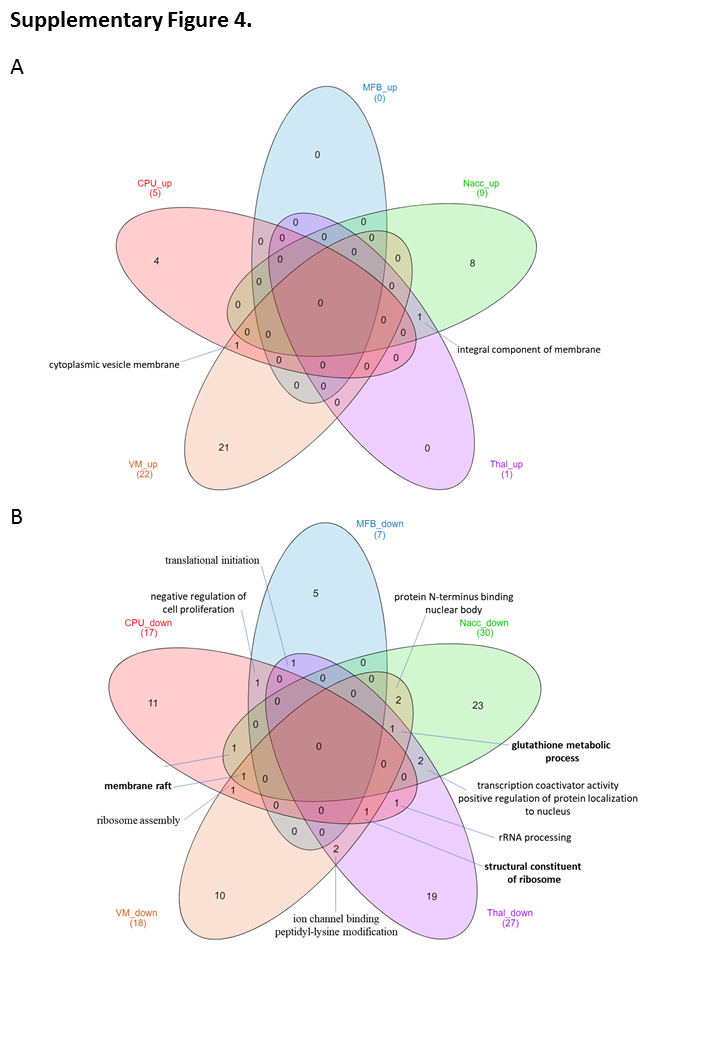

Supplement: Supplementary file 11 [file Image_4.TIF]

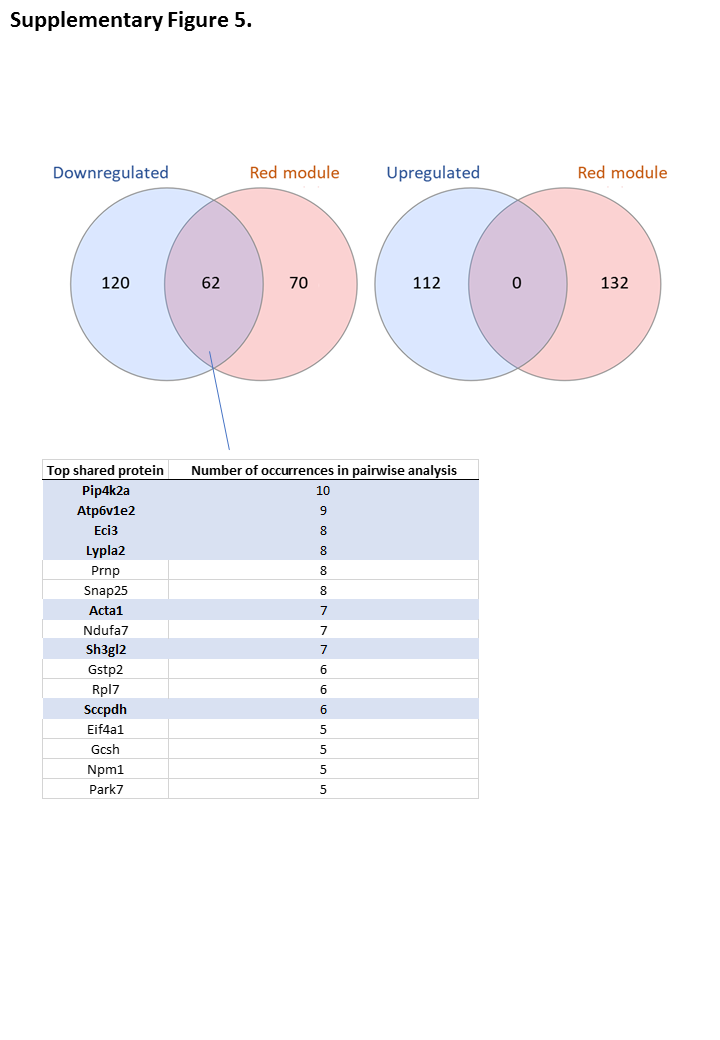

Supplement: Supplementary file 12 [file Image_5.TIF]
